# Crystal structure of ethyl 2-(5-amino-1-benzene­sulfonyl-3-oxo-2,3-di­hydro-1*H*-pyrazol-2-yl)acetate

**DOI:** 10.1107/S2056989020002674

**Published:** 2020-03-03

**Authors:** Nadia H. Metwally, Galal H. Elgemeie, Peter G. Jones

**Affiliations:** a Chemistry Department, Faculty of Science, Cairo University, Giza, Egypt; b Chemistry Department, Faculty of Science, Helwan University, Cairo, Egypt; c Institut für Anorganische und Analytische Chemie, Technische Universität Braunschweig, Hagenring 30, D-38106 Braunschweig, Germany

**Keywords:** crystal structure, pyrazine, sulfon­yl, hydrogen bonds

## Abstract

In the title compound, C_13_H_15_N_3_O_5_S, the two rings face each other in a ‘V′ form at the S atom, with one N—H⋯O=S and one C—H⋯O=S contact from the pyrazolyl substituents to the sulfonyl group. Two classical hydrogen bonds from the amine group, one of the form N—H⋯O=S and one N—H⋯O=C_oxo_, link the mol­ecules to form layers parallel to the *bc* plane.

## Chemical context   

We are inter­ested in the development of innovative synthetic strategies for *N*-sulfonyl- and *N*-sulfonyl­amino-based heterocyclic ring systems that have found application as new anti­microbial and anti-viral agents (Azzam *et al.*, 2017 ▸, 2019*a* ▸ *b* ▸; Elgemeie *et al.*, 2017 ▸, 2019 ▸; Zhu *et al.*, 2013 ▸). Michael *et al.* (2007 ▸) investigated the inhibition capabilities of a novel series of our reported *N*-sulfonyl­pyrazoles (Elgemeie *et al.*, 1998 ▸, 1999 ▸, 2013 ▸) towards the enzyme cathepsin B16. Shyama *et al.* (2009 ▸) also identified some of our reported *N*-aryl­sulfonyl­pyrazole series to be active inhibitors of the NS2B-NS3 virus. These promising results led our research group to investigate new approaches to other derivatives of *N*-sulfonyl­pyrazoles, thereby seeking alternative scaffolds for use as promising chemotherapeutics (Azzam & Elgemeie, 2019 ▸; Elgemeie & Jones, 2002 ▸; Zhang *et al.*, 2020 ▸). Accordingly, we synthesized the N-1-substituted derivative of *N*-sulfonyl­pyrazole **1**.
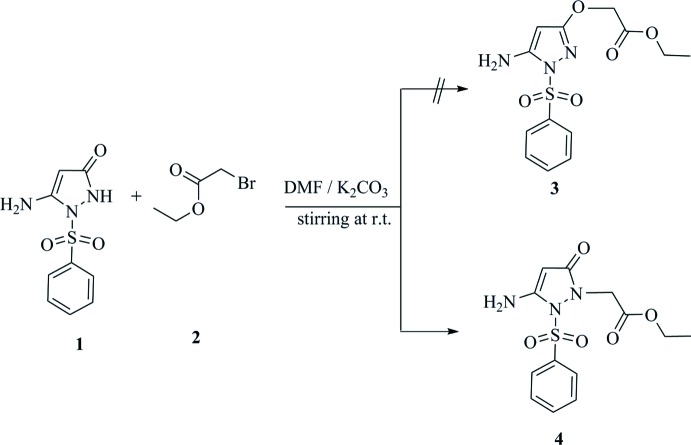


The reaction **1** with ethyl bromo­acetate **2** in the presence of anhydrous potassium carbonate in dry *N*,*N*-di­methyl­formamide at room temperature produced an adduct for which two possible isomers, the *O*-alkyl­ated or *N*-alkyl­ated *N*-sulfonyl­pyrazole structures **3** or **4**, were considered. The ^1^H NMR spectra of the product revealed the presence of an amino group at 7.34 ppm and a pyrazole CH at 4.34 ppm, but spectroscopic data cannot differentiate between structures **3** and **4**. The crystal structure determination indicated unambiguously the formation of the *N*-alkyl­ated *N*-sulfonyl­pyrazole **4** as the only product in the solid state.

## Structural commentary   

**Figure 1:**
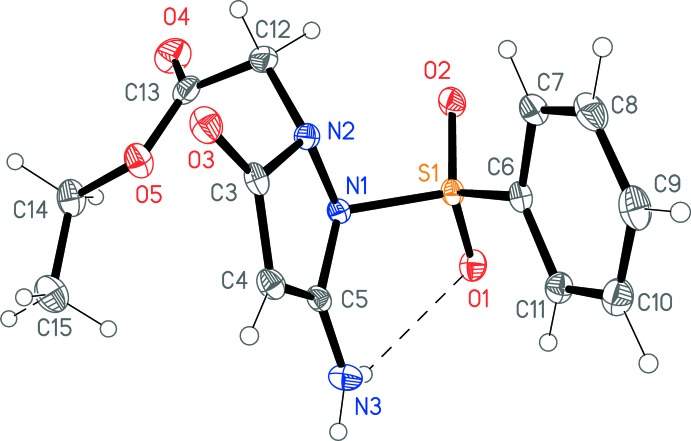
Structure of the title compound **4** in the crystal. Ellipsoids represent 50% probability levels. The dashed line indicates the intra­molecular hydrogen bond.

The structure analysis confirms the formation of compound **4** (Fig. 1 ▸). The mol­ecule displays an intra­molecular hydrogen bond of the form N—H⋯O=S, and the intra­molecular contact H12*A*⋯O2 is also quite short at 2.38 Å (Table 1 ▸). Accordingly, the two rings face each other in a roughly ‘V-shaped’ form around the central SO_2_ unit, with an inter­planar angle of 53.45 (5)° and torsion angles C7—C6⋯N1—N2 = −13.10 (10) and C11—C6⋯N1—C5 = 21.26 (11)°. The corresponding angle N1—S1—C6 is the narrowest at S1 (the largest is, as expected, O1=S=O2). In the pyrazole ring, the bond C4—C5 is the shortest, consistent with a major contribution from the resonance form shown in the Scheme. The exocyclic C5—N3 bond is appreciably shorter than the two C—N bonds in the ring. The side-chain atom sequence C12—C13—O5—C14—C15 displays an extended conformation. See Table 2 ▸ for selected mol­ecular dimensions.

## Supra­molecular features   

**Figure 2:**
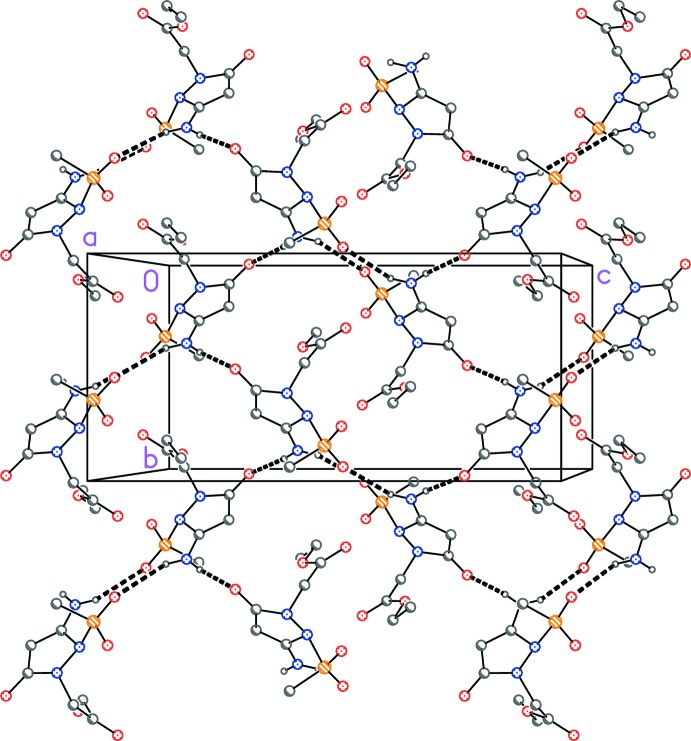
Packing diagram of **4** projected parallel to the *bc* plane. Dashed lines indicate inter­molecular hydrogen bonds (intra­molecular H bonds are omitted). Hydrogen atoms not involved in this hydrogen bonding system are omitted.

Two classical hydrogen bonds (Table 1 ▸) are observed, one from each hydrogen atom of the amino group; the contact H01⋯O1^i^, involving the same hydrogen atom that forms the intra­molecular hydrogen bond, is however much longer than H02⋯O3^ii^. The mol­ecules are thereby connected to form layers parallel to the *bc* plane (Fig. 2 ▸).

## Database survey   

A search of the Cambridge Database (Version 5.4; Groom *et al.*, 2016 ▸) for the fragment Ar-SO_2_ bonded to one nitro­gen atom of an NNCCC ring (all atoms three-coordinate, any bond orders and any or no other substituents) gave only two hits, our previously reported structures NARCOY (Ar = Ph; Elgemeie *et al.*, 1998 ▸) and LERBIV (Ar = *p*-Tol; Elgemeie *et al.*, 2013 ▸). These are closely related, but the former is pseudosymmetric; for a detailed discussion, see Elgemeie *et al.* (2013 ▸). Both bear the same oxo and amino substituents as in the current structure; the latter is, however, substituted at N2, so that one fewer hydrogen-bond donor is available and the packing is different from those of the previous structures.

## Synthesis and crystallization   

A mixture of compound **1** (0.01 mol), ethyl bromo­acetate **2** (0.01 mol) and anhydrous potassium carbonate (0.01 mol) in *N*,*N*-di­methyl­formamide (5 mL) was stirred at room temperature for 2 h. The mixture was poured onto ice–water; the solid thus formed was filtered off and recrystallized from ethanol to give pale yellow crystals in 60% yield, m.p. = 394 K. IR (KBr, cm^−1^): ν 3330, 3250 (NH_2_), 1730 (ester C=O), 1690 (ring C=O); ^1^H NMR (DMSO-*d*_6_): δ = 1.17 (*t*, 3H, *J* = 7.2 Hz, CH_3_), 4.07 (*q*, 2H, *J* = 7.2 Hz, CH_2_), 4.34 (*s*, 1H, CH), 4.43 (*s*, 2H, CH_2_), 7.34 (*s*, 2H, NH_2_), 7.63–7.88 (*m*, 5H, Ar). Analysis calculated C_13_H_15_N_3_O_5_S (325.34); C, 47.99; H, 4.65; N, 12.92; S, 9.85. Found: C, 48.17; H, 4.84; N, 13.15; S, 9.67%.

## Refinement   

Crystal data, data collection and structure refinement details are summarized in Table 3 ▸. The NH hydrogen atoms were refined freely. The methyl group was refined as an idealized rigid group allowed to rotate but not tip (‘AFIX 137′; C—H 0.98 Å, H—C—H 109.5°). Other hydrogen atoms were included using a riding model starting from calculated positions (C—H_aromatic_ = 0.95, C—H_methyl­ene_ = 0.99 Å). The *U*(H) values were fixed at 1.5 (for the methyl H) or 1.2 times the equivalent *U*_iso_ value of the parent carbon atoms.

## Supplementary Material

Crystal structure: contains datablock(s) I, global. DOI: 10.1107/S2056989020002674/nr2077sup1.cif

Structure factors: contains datablock(s) I. DOI: 10.1107/S2056989020002674/nr2077Isup2.hkl

Supporting information file. DOI: 10.1107/S2056989020002674/nr2077Isup3.cml

CCDC reference: 1986369

Additional supporting information:  crystallographic information; 3D view; checkCIF report

## Figures and Tables

**Table 1:** Hydrogen-bond geometry (Å, °)

*D*—H⋯*A*	*D*—H	H⋯*A*	*D*⋯*A*	*D*—H⋯*A*
N3—H01⋯O1	0.866 (19)	2.355 (19)	2.8296 (15)	114.8 (15)
N3—H01⋯O1^i^	0.866 (19)	2.593 (19)	3.3644 (15)	148.8 (16)
N3—H02⋯O3^ii^	0.871 (19)	1.961 (19)	2.8257 (15)	171.5 (17)
C12—H12*A*⋯O2	0.99	2.38	3.0214 (16)	122

Symmetry codes: (i) 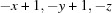; (ii) 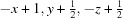.

**Table 2:** Selected geometric parameters (Å, °)

N1—C5	1.4305 (15)	N3—C5	1.3306 (16)
N1—N2	1.4313 (14)	C3—C4	1.4184 (18)
N2—C3	1.4139 (15)	C4—C5	1.3640 (17)
			
O2—S1—O1	120.63 (6)	N1—S1—C6	104.30 (5)
			
C14—O5—C13—C12	−175.81 (11)	C13—O5—C14—C15	158.95 (12)

**Table 3:** Experimental details

Crystal data	
Chemical formula	C_13_H_15_N_3_O_5_S
*M*_r_	325.34
Crystal system, space group	Monoclinic, *P*2_1_/*c*
Temperature (K)	100
*a*, *b*, *c* (Å)	9.2139 (4), 8.8122 (4), 18.3486 (7)
β (°)	104.521 (4)
*V* (Å^3^)	1442.22 (11)
*Z*	4
Radiation type	Mo *K*α
μ (mm^−1^)	0.25
Crystal size (mm)	0.35 × 0.30 × 0.15

Data collection	
Diffractometer	Oxford Diffraction Xcalibur Eos
Absorption correction	Multi-scan (*CrysAlis PRO*; Rigaku OD, 2015 ▸)
*T*_min_, *T*_max_	0.964, 1.000
No. of measured, independent and observed [*I* > 2σ(*I*)] reflections	74051, 4193, 3708
*R*_int_	0.044
(sin θ/λ)_max_ (Å^−1^)	0.704

Refinement	
*R*[*F* ^2^ > 2σ(*F* ^2^)], *wR*(*F* ^2^), *S*	0.035, 0.085, 1.11
No. of reflections	4193
No. of parameters	208
H-atom treatment	H atoms treated by a mixture of independent and constrained refinement
Δρ_max_, Δρ_min_ (e Å^−3^)	0.47, −0.31

Computer programs: *CrysAlis PRO* (Rigaku OD, 2015 ▸), *SHELXS97* (Sheldrick, 2008 ▸), *SHELXL2017* (Sheldrick, 2015 ▸) and *XP* (Siemens, 1994 ▸).

## supplementary crystallographic information

### Crystal data

**Table:**

C_13_H_15_N_3_O_5_S	*F*(000) = 680
*M**_r_* = 325.34	*D*_x_ = 1.498 Mg m^−^^3^
Monoclinic, *P*2_1_/*c*	Mo *K*α radiation, λ = 0.71073 Å
*a* = 9.2139 (4) Å	Cell parameters from 16307 reflections
*b* = 8.8122 (4) Å	θ = 2.6–30.3°
*c* = 18.3486 (7) Å	µ = 0.25 mm^−^^1^
β = 104.521 (4)°	*T* = 100 K
*V* = 1442.22 (11) Å^3^	Tablet, colourless
*Z* = 4	0.35 × 0.30 × 0.15 mm

### Data collection

**Table:**

Oxford Diffraction Xcalibur Eos diffractometer	4193 independent reflections
Radiation source: fine-focus sealed X-ray tube	3708 reflections with *I* > 2σ(*I*)
Detector resolution: 16.1419 pixels mm^-1^	*R*_int_ = 0.044
ω–scan	θ_max_ = 30.0°, θ_min_ = 2.3°
Absorption correction: multi-scan (CrysAlis PRO; Rigaku OD, 2015)	*h* = −12→12
*T*_min_ = 0.964, *T*_max_ = 1.000	*k* = −12→12
74051 measured reflections	*l* = −25→25

### Refinement

**Table:**

Refinement on *F*^2^	Primary atom site location: structure-invariant direct methods
Least-squares matrix: full	Secondary atom site location: difference Fourier map
*R*[*F*^2^ > 2σ(*F*^2^)] = 0.035	Hydrogen site location: mixed
*wR*(*F*^2^) = 0.085	H atoms treated by a mixture of independent and constrained refinement
*S* = 1.11	*w* = 1/[σ^2^(*F*_o_^2^) + (0.0304*P*)^2^ + 0.8673*P*] where *P* = (*F*_o_^2^ + 2*F*_c_^2^)/3
4193 reflections	(Δ/σ)_max_ < 0.001
208 parameters	Δρ_max_ = 0.47 e Å^−^^3^
0 restraints	Δρ_min_ = −0.31 e Å^−^^3^

### Special details

**Table:**

Geometry. All esds (except the esd in the dihedral angle between two l.s. planes) are estimated using the full covariance matrix. The cell esds are taken into account individually in the estimation of esds in distances, angles and torsion angles; correlations between esds in cell parameters are only used when they are defined by crystal symmetry. An approximate (isotropic) treatment of cell esds is used for estimating esds involving l.s. planes.
Refinement. The NH hydrogens were refined freely. The methyl was refined as an idealized rigid group allowed to rotate but not tip. Other hydrogens were included using a riding model starting from calculated positions.

### Fractional atomic coordinates and isotropic or equivalent isotropic displacement parameters (Å^2^)

**Table:**

	*x*	*y*	*z*	*U*_iso_*/*U*_eq_	
S1	0.21827 (3)	0.35269 (3)	0.02872 (2)	0.01371 (8)	
N1	0.33782 (11)	0.22191 (12)	0.08270 (6)	0.01304 (19)	
N2	0.25215 (11)	0.11750 (12)	0.11476 (6)	0.0142 (2)	
N3	0.54212 (13)	0.38935 (14)	0.13239 (7)	0.0187 (2)	
H01	0.547 (2)	0.411 (2)	0.0871 (11)	0.029 (5)*	
H02	0.602 (2)	0.431 (2)	0.1716 (11)	0.027 (4)*	
O1	0.31374 (10)	0.45322 (11)	0.00110 (5)	0.01845 (19)	
O2	0.10901 (10)	0.26285 (11)	−0.02197 (5)	0.01864 (19)	
O3	0.27967 (10)	0.00832 (11)	0.23139 (5)	0.0201 (2)	
O4	0.32627 (13)	−0.17922 (12)	−0.00343 (6)	0.0282 (2)	
O5	0.46016 (11)	−0.09414 (11)	0.10922 (5)	0.0206 (2)	
C3	0.32177 (13)	0.10511 (15)	0.19250 (7)	0.0151 (2)	
C4	0.43781 (13)	0.21500 (15)	0.20999 (7)	0.0159 (2)	
H4	0.497420	0.238013	0.258957	0.019*	
C5	0.44943 (13)	0.28208 (14)	0.14470 (7)	0.0139 (2)	
C6	0.13579 (13)	0.44475 (14)	0.09267 (7)	0.0148 (2)	
C7	0.00733 (14)	0.38207 (15)	0.10759 (7)	0.0187 (2)	
H7	−0.038852	0.294967	0.081123	0.022*	
C8	−0.05126 (15)	0.45024 (17)	0.16212 (8)	0.0219 (3)	
H8	−0.137945	0.408731	0.173735	0.026*	
C9	0.01580 (15)	0.57863 (17)	0.19982 (8)	0.0223 (3)	
H9	−0.025685	0.624603	0.236866	0.027*	
C10	0.14298 (15)	0.64049 (16)	0.18388 (7)	0.0206 (3)	
H10	0.187588	0.728962	0.209625	0.025*	
C11	0.20494 (14)	0.57298 (15)	0.13031 (7)	0.0173 (2)	
H11	0.292829	0.613492	0.119539	0.021*	
C12	0.20730 (14)	−0.01978 (15)	0.07046 (7)	0.0189 (2)	
H12A	0.136124	0.008368	0.022367	0.023*	
H12B	0.153689	−0.087254	0.098087	0.023*	
C13	0.33651 (15)	−0.10706 (15)	0.05324 (7)	0.0185 (2)	
C14	0.59637 (16)	−0.16389 (17)	0.09781 (8)	0.0225 (3)	
H14A	0.602432	−0.149340	0.045151	0.027*	
H14B	0.596692	−0.274125	0.108255	0.027*	
C15	0.72666 (16)	−0.08824 (19)	0.15109 (8)	0.0263 (3)	
H15A	0.724485	0.020857	0.140513	0.039*	
H15B	0.820526	−0.131624	0.144566	0.039*	
H15C	0.720047	−0.104563	0.202995	0.039*	

### Atomic displacement parameters (Å^2^)

**Table:**

	*U*^11^	*U*^22^	*U*^33^	*U*^12^	*U*^13^	*U*^23^
S1	0.01461 (14)	0.01428 (15)	0.01147 (13)	0.00241 (10)	0.00185 (10)	0.00165 (10)
N1	0.0123 (4)	0.0133 (5)	0.0126 (4)	0.0006 (4)	0.0014 (3)	0.0012 (4)
N2	0.0141 (5)	0.0141 (5)	0.0136 (4)	−0.0002 (4)	0.0021 (4)	0.0024 (4)
N3	0.0174 (5)	0.0223 (6)	0.0158 (5)	−0.0045 (4)	0.0029 (4)	−0.0011 (4)
O1	0.0216 (4)	0.0181 (5)	0.0170 (4)	0.0021 (4)	0.0074 (3)	0.0038 (3)
O2	0.0191 (4)	0.0197 (5)	0.0137 (4)	0.0022 (4)	−0.0021 (3)	−0.0003 (3)
O3	0.0190 (4)	0.0245 (5)	0.0177 (4)	0.0022 (4)	0.0060 (3)	0.0066 (4)
O4	0.0372 (6)	0.0253 (5)	0.0202 (5)	−0.0032 (4)	0.0037 (4)	−0.0071 (4)
O5	0.0211 (5)	0.0230 (5)	0.0167 (4)	0.0060 (4)	0.0029 (3)	−0.0030 (4)
C3	0.0134 (5)	0.0183 (6)	0.0136 (5)	0.0055 (4)	0.0035 (4)	0.0017 (4)
C4	0.0150 (5)	0.0199 (6)	0.0120 (5)	0.0025 (5)	0.0017 (4)	−0.0010 (4)
C5	0.0112 (5)	0.0154 (6)	0.0144 (5)	0.0025 (4)	0.0019 (4)	−0.0025 (4)
C6	0.0145 (5)	0.0155 (6)	0.0143 (5)	0.0042 (4)	0.0033 (4)	0.0020 (4)
C7	0.0147 (5)	0.0191 (6)	0.0210 (6)	0.0019 (5)	0.0023 (5)	0.0017 (5)
C8	0.0157 (6)	0.0273 (7)	0.0243 (6)	0.0040 (5)	0.0078 (5)	0.0047 (5)
C9	0.0226 (6)	0.0265 (7)	0.0187 (6)	0.0086 (5)	0.0070 (5)	0.0018 (5)
C10	0.0238 (6)	0.0186 (6)	0.0186 (6)	0.0035 (5)	0.0039 (5)	−0.0007 (5)
C11	0.0177 (6)	0.0155 (6)	0.0184 (6)	0.0016 (5)	0.0039 (4)	0.0020 (5)
C12	0.0183 (6)	0.0160 (6)	0.0194 (6)	−0.0026 (5)	−0.0010 (5)	0.0006 (5)
C13	0.0250 (6)	0.0130 (6)	0.0163 (6)	−0.0026 (5)	0.0029 (5)	0.0018 (4)
C14	0.0249 (7)	0.0235 (7)	0.0206 (6)	0.0084 (5)	0.0086 (5)	−0.0015 (5)
C15	0.0221 (6)	0.0333 (8)	0.0242 (7)	0.0047 (6)	0.0074 (5)	0.0012 (6)

### Geometric parameters (Å, º)

**Table:**

S1—O2	1.4268 (9)	C6—C7	1.3941 (17)
S1—O1	1.4280 (10)	C7—C8	1.3871 (19)
S1—N1	1.7249 (10)	C7—H7	0.9500
S1—C6	1.7491 (12)	C8—C9	1.388 (2)
N1—C5	1.4305 (15)	C8—H8	0.9500
N1—N2	1.4313 (14)	C9—C10	1.388 (2)
N2—C3	1.4139 (15)	C9—H9	0.9500
N2—C12	1.4583 (16)	C10—C11	1.3883 (18)
N3—C5	1.3306 (16)	C10—H10	0.9500
N3—H01	0.866 (19)	C11—H11	0.9500
N3—H02	0.871 (19)	C12—C13	1.5156 (19)
O3—C3	1.2353 (16)	C12—H12A	0.9900
O4—C13	1.2023 (16)	C12—H12B	0.9900
O5—C13	1.3338 (16)	C14—C15	1.501 (2)
O5—C14	1.4589 (16)	C14—H14A	0.9900
C3—C4	1.4184 (18)	C14—H14B	0.9900
C4—C5	1.3640 (17)	C15—H15A	0.9800
C4—H4	0.9500	C15—H15B	0.9800
C6—C11	1.3926 (18)	C15—H15C	0.9800
			
O2—S1—O1	120.63 (6)	C7—C8—H8	119.8
O2—S1—N1	104.37 (5)	C9—C8—H8	119.8
O1—S1—N1	104.88 (5)	C8—C9—C10	120.59 (12)
O2—S1—C6	109.90 (6)	C8—C9—H9	119.7
O1—S1—C6	111.12 (6)	C10—C9—H9	119.7
N1—S1—C6	104.30 (5)	C11—C10—C9	119.97 (13)
C5—N1—N2	105.78 (9)	C11—C10—H10	120.0
C5—N1—S1	115.93 (8)	C9—C10—H10	120.0
N2—N1—S1	109.08 (7)	C10—C11—C6	118.73 (12)
C3—N2—N1	107.87 (9)	C10—C11—H11	120.6
C3—N2—C12	119.46 (10)	C6—C11—H11	120.6
N1—N2—C12	114.36 (10)	N2—C12—C13	114.18 (10)
C5—N3—H01	120.9 (12)	N2—C12—H12A	108.7
C5—N3—H02	117.4 (12)	C13—C12—H12A	108.7
H01—N3—H02	121.5 (17)	N2—C12—H12B	108.7
C13—O5—C14	116.94 (10)	C13—C12—H12B	108.7
O3—C3—N2	120.48 (12)	H12A—C12—H12B	107.6
O3—C3—C4	131.97 (12)	O4—C13—O5	125.29 (13)
N2—C3—C4	107.53 (10)	O4—C13—C12	123.59 (12)
C5—C4—C3	108.53 (11)	O5—C13—C12	111.11 (11)
C5—C4—H4	125.7	O5—C14—C15	107.20 (11)
C3—C4—H4	125.7	O5—C14—H14A	110.3
N3—C5—C4	130.69 (12)	C15—C14—H14A	110.3
N3—C5—N1	119.50 (11)	O5—C14—H14B	110.3
C4—C5—N1	109.80 (11)	C15—C14—H14B	110.3
C11—C6—C7	121.97 (12)	H14A—C14—H14B	108.5
C11—C6—S1	119.17 (9)	C14—C15—H15A	109.5
C7—C6—S1	118.76 (10)	C14—C15—H15B	109.5
C8—C7—C6	118.25 (12)	H15A—C15—H15B	109.5
C8—C7—H7	120.9	C14—C15—H15C	109.5
C6—C7—H7	120.9	H15A—C15—H15C	109.5
C7—C8—C9	120.49 (12)	H15B—C15—H15C	109.5
			
O2—S1—N1—C5	−172.95 (9)	O2—S1—C6—C11	−159.28 (10)
O1—S1—N1—C5	59.29 (9)	O1—S1—C6—C11	−23.16 (12)
C6—S1—N1—C5	−57.61 (10)	N1—S1—C6—C11	89.33 (10)
O2—S1—N1—N2	−53.74 (9)	O2—S1—C6—C7	24.42 (12)
O1—S1—N1—N2	178.50 (8)	O1—S1—C6—C7	160.54 (10)
C6—S1—N1—N2	61.60 (9)	N1—S1—C6—C7	−86.97 (10)
C5—N1—N2—C3	−5.93 (12)	C11—C6—C7—C8	−0.47 (19)
S1—N1—N2—C3	−131.28 (8)	S1—C6—C7—C8	175.72 (10)
C5—N1—N2—C12	−141.43 (10)	C6—C7—C8—C9	0.85 (19)
S1—N1—N2—C12	93.23 (10)	C7—C8—C9—C10	−0.3 (2)
N1—N2—C3—O3	−171.44 (11)	C8—C9—C10—C11	−0.6 (2)
C12—N2—C3—O3	−38.61 (17)	C9—C10—C11—C6	0.97 (19)
N1—N2—C3—C4	7.23 (13)	C7—C6—C11—C10	−0.44 (19)
C12—N2—C3—C4	140.06 (11)	S1—C6—C11—C10	−176.61 (10)
O3—C3—C4—C5	172.70 (13)	C3—N2—C12—C13	−74.54 (14)
N2—C3—C4—C5	−5.75 (14)	N1—N2—C12—C13	55.45 (14)
C3—C4—C5—N3	−179.13 (13)	C14—O5—C13—O4	5.0 (2)
C3—C4—C5—N1	2.02 (14)	C14—O5—C13—C12	−175.81 (11)
N2—N1—C5—N3	−176.57 (11)	N2—C12—C13—O4	−147.96 (13)
S1—N1—C5—N3	−55.56 (13)	N2—C12—C13—O5	32.79 (15)
N2—N1—C5—C4	2.43 (13)	C13—O5—C14—C15	158.95 (12)
S1—N1—C5—C4	123.44 (10)		

### Hydrogen-bond geometry (Å, º)

**Table:**

*D*—H···*A*	*D*—H	H···*A*	*D*···*A*	*D*—H···*A*
N3—H01···O1	0.866 (19)	2.355 (19)	2.8296 (15)	114.8 (15)
N3—H01···O1^i^	0.866 (19)	2.593 (19)	3.3644 (15)	148.8 (16)
N3—H02···O3^ii^	0.871 (19)	1.961 (19)	2.8257 (15)	171.5 (17)
C12—H12*A*···O2	0.99	2.38	3.0214 (16)	122

Symmetry codes: (i) −*x*+1, −*y*+1, −*z*; (ii) −*x*+1, *y*+1/2, −*z*+1/2.
